# Circulating Tumor DNA and Circulating Tumor Cells in Liquid Biopsy as Post-Treatment Prognostic Biomarkers in Early Breast Cancer: A Systematic Review and Meta-Analysis

**DOI:** 10.3390/ijms27177719

**Published:** 2026-08-28

**Authors:** Einas M. Yousef, Motaz Talaat Elghnam, Hiba Elhassan, Malak Hassan, Saifeldin Yousef, Hend Aabed, Noha Mitwally

**Affiliations:** 1 Department of Anatomy & Genetics, College of Medicine, Alfaisal University, Riyadh 11533, Saudi Arabia; melghnam@alfaisal.edu; 2 Anatomy and Embryology Department, Faculty of Medicine, Cairo University, Cairo 11956, Egypt; 3 College of Medicine, Alfaisal University, Riyadh 11533, Saudi Arabia; helhassan@alfaisal.edu (H.E.); mhhassan@alfaisal.edu (M.H.); 4 Faculty of Medicine, Badr University in Cairo, Cairo 11829, Egypt; saifeldin.2023000561@buc.edu.eg; 5 Department of Anatomic Pathology, Cairo University Hospitals, Cairo 11956, Egypt; hendaabed@gmail.com; 6 Department of Biochemistry and Molecular Biology, Faculty of Pharmacy, Cairo University, Cairo 11562, Egypt; nohamtwally@gmail.com

**Keywords:** circulating tumor cells, circulating tumor DNA, minimal residual disease, early breast cancer, liquid biopsy, prognostic biomarker, systematic review, meta-analysis

## Abstract

Despite advances in systemic therapy, many early breast cancer patients experience recurrence due to subclinical minimal residual disease (MRD). Circulating tumor DNA (ctDNA) and circulating tumor cells (CTCs) have emerged as promising liquid biopsy markers for post-treatment MRD detection. This pre-registered systematic review and meta-analysis (PROSPERO/PRISMA 2020) evaluated their comparative prognostic significance. Seven databases were searched from inception to March 2026. Eligible studies included early breast cancer patients undergoing post-treatment ctDNA or CTC assessment after neoadjuvant or adjuvant therapy, reporting survival outcomes with extractable hazard ratios. Quality was assessed using the QUIPS tool; random-effects meta-analyses used the REML estimator. Seventeen studies (thirteen ctDNA, four CTCs; n = 3030) were included. Post-treatment CTC positivity was significantly associated with poorer survival (pooled HR = 2.99, 95% CI: 1.99–4.49; I^2^ = 17.2%). ctDNA positivity demonstrated a substantially stronger prognostic effect (pooled HR = 10.28, 95% CI: 6.32–16.70; I^2^ = 47.3%). Subgroup analyses identified assessment timing as a key heterogeneity source, with stronger effects after adjuvant (HR = 20.62; k = 4) versus neoadjuvant therapy (HR = 6.07; k = 9); given the small number of post-adjuvant studies, this finding is hypothesis-generating. No significant publication bias was detected. Both markers were significant prognostic markers of MRD. ctDNA showed a stronger pooled prognostic association, although no study assessed both biomarkers within the same cohort and direct head-to-head comparisons therefore remain lacking. Prospective randomized trials are needed to evaluate MRD-guided treatment strategies.

## 1. Introduction

Breast cancer remains the most commonly diagnosed malignancy globally, with an incidence rate of 46.8 per 100,000 women worldwide and over 2.3 million new cases annually [1,2]. It is also considered the leading cause of cancer-related mortality in women [1]. Early breast cancer broadly means breast malignancy that is still limited to the breast and related lymph nodes, without dissemination to distant organs [3]. It is also described in other references as tumors of up to approximately 5 cm in size with no distant metastases [4]. In early breast cancer, surgery combined with systemic neoadjuvant or adjuvant therapy has dramatically improved survival outcomes. However, between 20% and 30% of patients with early BC experience disease recurrence despite completion of multimodal treatment [5,6]. 

Current clinicopathological prognostic factors, including tumor stage, nodal status, histological grade, hormonal receptor status, and genomic signatures, help estimate population-level risk stratification but remain less accurate for predicting outcomes in individual patients [7,8]. Pathological complete response (pCR) following neoadjuvant therapy is considered a surrogate for long-term outcomes in triple-negative and Human Epidermal Growth Factor Receptor 2 (HER2)-positive breast cancer [9]. However, a substantial proportion of patients achieving pCR still experience relapse, while some with residual disease remain disease-free for many years [10,11]. These limitations highlight the urgent need for sensitive, real-time biomarkers capable of detecting and monitoring subclinical residual disease at the molecular level. Hence, clinical attention has shifted toward minimal residual disease (MRD), which is believed to play a central role in late breast cancer relapse [12].

MRD refers to the small amount of residual tumor burden that remains after treatment and is below the detection limit of conventional imaging or routine pathological examination [13]. Liquid biopsy is a minimally invasive approach that detects cancer-associated material circulating in peripheral blood. Two principal approaches of liquid biopsy analysis have been evaluated in early breast cancer: circulating tumor cells (CTCs) and circulating tumor DNA (ctDNA) [14,15]. CTCs are intact, viable cancer cells shed from the primary tumor or metastatic sites into the bloodstream and are detectable by immunocytochemical or molecular techniques. The detection of CTCs in peripheral blood after definitive treatment suggests the persistence of viable cancer cells that have evaded systemic therapy and may initiate future metastases [16]. In contrast, ctDNA consists of small fragments of DNA released from apoptotic or necrotic tumor cells into the bloodstream, carrying tumor-specific genetic or epigenetic alterations that distinguish them from normal circulating cell-free DNA [17]. Both CTCs and ctDNAs are promising MRD biomarkers, offering the possibility for the early detection of recurrence, real-time treatment monitoring, and more personalized risk stratification. 

Despite their shared clinical application, CTCs and ctDNA differ fundamentally in their biological characteristics, detection methodologies, and potential clinical utility. CTCs represent the cellular component of MRD and may provide information about tumor phenotype, epithelial-to-mesenchymal transition, and mechanisms of treatment resistance [18]. However, they are typically present at low frequencies in peripheral blood and require specialized detection techniques. In contrast, ctDNA can be detected at significantly lower tumor burdens and generally offers higher analytical sensitivity through modern, personalized, tumor-informed assays [19,20]. However, ctDNA provides only genetic information and cannot assess the behavior or viability of cancer cells. Characterizing the prognostic performance of each biomarker and the clinical circumstances in which each is informative is essential for designing future MRD-guided clinical trials.

Previous systematic reviews have focused on either CTCs or ctDNA, have inconsistently pooled univariable and multivariable analyses, and have not addressed platform heterogeneity or evaluated the two biomarkers within a single analytical framework [21,22,23,24]. We therefore conducted a pre-registered, PRISMA-compliant systematic review and meta-analysis to quantify the pooled prognostic effect of post-treatment ctDNA and CTCs on survival outcomes in early breast cancer and to identify clinically relevant moderators of these associations through pre-specified subgroup analyses.

## 2. Materials and Methods

### 2.1. Protocol Registration and Reporting 

This systematic review and meta-analysis was prospectively registered with the International Prospective Register of Systematic Reviews (PROSPERO; CRD420261330223). The study was conducted and reported in accordance with the Preferred Reporting Items for Systematic Reviews and Meta-Analyses (PRISMA) 2020 guidelines [25]. 

### 2.2. Eligibility Criteria 

Studies were eligible for inclusion if they satisfied all of the following criteria: (1) Including female participants with early-stage (non-metastatic) breast cancer; (2) CTCs or ctDNA measured in blood at a clearly defined post-treatment timepoint following completion of neoadjuvant chemotherapy (NACT), adjuvant chemotherapy (adj CT), or adjuvant endocrine therapy (NET); (3) studies reported MRD-negative vs MRD-positive within the same cohort, where MRD positivity was defined as detection of ctDNA or CTCs according to each study’s predefined threshold; (4) survival outcomes reported, including disease-free survival (DFS), recurrence-free survival (RFS), event-free survival (EFS), distant recurrence-free survival/interval (DRFS/DRFI), or overall survival (OS); (5) hazard ratios (HRs) with 95% confidence intervals (CIs) reported from the published data; and (6) original research articles (prospective or retrospective cohort studies, randomized controlled trial secondary analyses, and observational studies reporting outcomes by MRD status). 

Studies were excluded if they did not specifically assess breast cancer patients undergoing adjuvant and/or neoadjuvant chemotherapy, included exclusively metastatic (stage IV) breast cancer or a mixed population where early breast cancer could not be separated, assessed only baseline or pre-treatment liquid biopsy, or reported serial monitoring without a fixed post-treatment MRD assessment timepoint, liquid biopsy not from blood (e.g., urine, bone marrow), or tumor tissue only. Moreover, reviews, case reports, conference abstracts without full-text data, animal studies, and studies that provided no extractable effect size data were excluded. Studies not in English were also excluded.

### 2.3. Search Strategy and Information Sources

Systematic electronic searches were conducted by H.E., M.H., and N.M. across MEDLINE/PubMed, EMBASE, Web of Science, Scopus, EBSCO, CINAHL, and the Cochrane Central Register of Controlled Trials from database inception to 31 March 2026. The search strategy combined controlled vocabulary terms (MeSH/EMTREE) with free-text keywords encompassing the following: breast cancer; circulating tumor cells; circulating tumor DNA; cell-free DNA; liquid biopsy; minimal residual disease; neoadjuvant; adjuvant; prognosis; disease-free survival; recurrence-free survival; hazard ratio. A complementary search of ClinicalTrials.gov was conducted to identify completed trials with relevant liquid-biopsy endpoints. Reference lists of identified systematic reviews and eligible studies were manually screened for additional records. All records were imported into Rayyan (Qatar Computing Research Institute) for study selection. Titles, abstracts, and full texts were screened independently by two reviewers, with disagreements resolved through discussion and consensus.

### 2.4. Data Extraction

Data were extracted independently by two reviewer teams (E.Y. and M.E.; S.Y. and H.A.) using a pre-specified standardized form. Disagreements were resolved through discussion and, when necessary, consultation of a third reviewer (N.M). Extracted data included the following: first author; year of publication; country; study design; sample size; breast cancer stage distribution; molecular subtype; liquid biopsy analyte (CTC or ctDNA); detection platform; post-treatment MRD assessment timepoint; CTC/ctDNA positivity cutoff definition; survival outcome; HR with 95% CI; analysis type (univariable (UV) or multivariable (MV)); adjustment variables (for MV analyses); and median follow-up duration. Importantly, multivariable HRs were preferentially selected when both MV and UV estimates were reported. UV estimates were included only when MV analyses were unavailable and were examined separately in sensitivity analyses. When multiple publications originated from the same patient cohort, overlap was assessed based on study center, recruitment period, patient characteristics, and author list. To avoid duplicate patient inclusion, only the most informative dataset (largest sample size, longest follow-up, or most comprehensive multivariable analysis) was included in the primary meta-analysis.

### 2.5. Quality Assessment

Risk of bias was assessed using the Quality In Prognosis Studies (QUIPS) tool, which was specifically developed for prognostic factor studies and is recommended by the Cochrane Prognosis Methods Group [26]. Each included study was evaluated across six domains, including study participation, study attrition, prognostic factor measurement, outcome measurement, study confounding, and statistical analysis and reporting. Each domain was rated as having a low, moderate, or high risk of bias according to the criteria specified in the original QUIPS guidance based on a detailed review of the full-text methods and results of each study. Particular attention was given to the domains most likely to influence prognostic estimates, namely prognostic factor measurement and study confounding. An overall risk-of-bias judgment was assigned to each study from the pattern of domain-level ratings. 

### 2.6. Statistical Analysis 

Separate meta-analyses were conducted for CTC and ctDNA studies. The primary outcome was the HR for the association between post-treatment liquid biopsy positivity and recurrence-related survival outcomes. Recurrence-related outcomes, including RFS, DFS, DFI, DRFS, DRFI, EFS, PFS, RFI, and metastatic recurrence, were pooled into a single recurrence outcome. Endpoint-specific analyses were not feasible because of the small number of eligible studies and the sparse and inconsistent reporting of individual endpoints. We acknowledge that these outcomes are not identical. Definitions varied across studies: composite endpoints such as EFS and DFS could include second primary malignancies and deaths from any cause, whereas recurrence-based endpoints (RFI and DRFI) focused more specifically on breast-cancer recurrence, and PFS was reported by only one study (Zaikova et al., 2024) [27]. The influence of individual studies, including the single study reporting PFS, was evaluated using leave-one-out sensitivity analysis (Section 3.4; Supplementary Table S1). Potential heterogeneity arising from differences in endpoint definitions was considered when interpreting the pooled estimates.

When multiple outcomes were reported, DFS, RFS, and EFS were prioritized for pooling. When multiple eligible post-treatment time points were reported, the HR corresponding to the pre-specified MRD assessment time point was selected. The primary analyses included all eligible studies with extractable HRs and 95% confidence intervals. Multivariable HRs were preferred whenever a study reported them, whereas univariable HRs were used only for the six studies that did not report adjusted models. Restricting the primary analysis to adjusted HRs, as recommended by several meta-analysis guidelines, would have excluded these six studies (46% of the ctDNA evidence base) and reduced the pool to seven studies for analysis. This would have reduced statistical precision and limited the ability to conduct the pre-specified subgroup and publication-bias analyses. Therefore, both adjusted and unadjusted HRs were included in the primary analysis. Adjustment status was evaluated in meta-regression and in the sensitivity analysis restricted to multivariable estimates. Because univariable HRs are not adjusted for established clinicopathological prognostic factors, the primary pooled estimate is interpreted as the overall prognostic association of ctDNA positivity rather than its independent prognostic effect.

Pooled HRs were calculated using the restricted maximum-likelihood (REML) random-effects model and inverse-variance weighting. Between-study heterogeneity was quantified using Cochran’s Q statistic (*p* < 0.10 as threshold for significance), the I^2^ statistic (interpreted as <25% low; 25–50% moderate; 50–75% high; >75% very high heterogeneity), and the between-study variance (τ^2^, REML estimator). Furthermore, pre-specified subgroup analyses were conducted for ctDNA studies according to the timing of assessment (post-neoadjuvant versus post-adjuvant therapy), breast cancer subtype, type of statistical adjustment (multivariable versus univariable analysis), and ctDNA detection methodology. For CTC studies, subgroup analyses were performed according to the timing of assessment and breast cancer subtype. Differences between subgroups were evaluated using mixed-effects meta-regression models, and the significance of moderators was assessed using the QM statistic.

Several sensitivity analyses included leave-one-out analyses, a comparison of random-effects and fixed-effects models, restriction to studies reporting multivariable-adjusted hazard ratios, and the exclusion of studies identified as major contributors to heterogeneity. Additional exploratory subgroup analyses compared ctDNA studies by assay design (tumor-informed versus tumor-agnostic), detection method (mutation-based versus methylation-based), and MRD-positivity definition (any detectable ctDNA or ≥1 variant versus ≥2 variants). For the CTC pool, a corresponding analysis compared the FDA-cleared CellSearch platform with the other detection systems. These four analyses were not pre-specified in the PROSPERO protocol and were therefore reported as exploratory. 

Publication bias was assessed for the ctDNA studies using funnel plot inspection and Egger’s regression test for funnel plot asymmetry (applicable when n ≥ 10). A formal assessment of publication bias was not performed for the CTC meta-analysis because fewer than ten studies were available. Trim-and-fill analysis was performed where indicated to estimate a publication-bias-adjusted pooled HR. All analyses were performed using R software (version R 4.6.0, R Foundation for Statistical Computing, Vienna, Austria) with the metafor package version 5.0.1. Statistical significance was defined as two-sided *p* < 0.05.

## 3. Results

### 3.1. Study Selection

**Figure 1:**
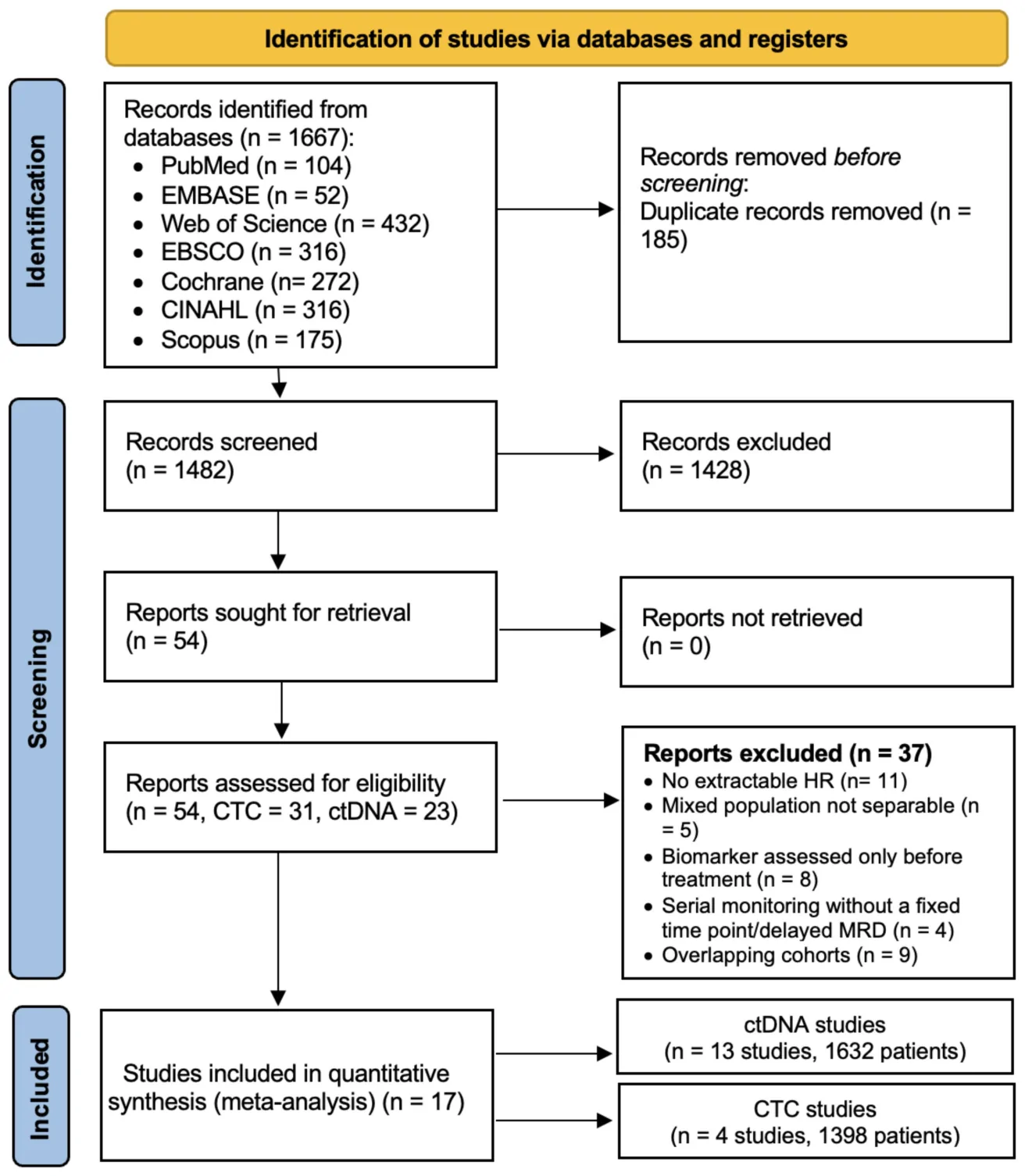
PRISMA flow diagram. CTC, circulating tumor cells; ctDNA, circulating tumor DNA; HR, hazard ratio; CI, confidence interval; MRD, minimal residual disease; NACT, neoadjuvant chemotherapy.

Systematic electronic searches across seven databases from inception to March 2026 (PubMed/MEDLINE, EMBASE, Web of Science, EBSCO, Cochrane Central Register of Controlled Trials, CINAHL, and Scopus) identified 1667 records (H.E., M.H., N.M.). Rayyan was used for the next steps of record screening. After the removal of 185 duplicates, 1482 unique records remained for title and abstract screening. Following independent screening by two reviewer teams (E.Y. and M.E.; S.Y. and H.A.), 1428 records were excluded for not meeting the eligibility criteria, and 54 full-text articles were retrieved for detailed eligibility assessment (31 CTC-related and 23 ctDNA-related studies). Any disagreements were resolved through discussion and consensus. A total of seventeen studies met all pre-specified eligibility criteria and were included in the quantitative synthesis, comprising four CTC studies (1398 patients) and thirteen ctDNA studies (1632 patients). Because the CTC literature in early breast cancer is considerably larger than the number of studies retained here, the CTC studies assessed in full text but excluded from the meta-analysis, together with the reasons for exclusion, are listed in Supplementary Table S2. The complete study selection process is summarized in the PRISMA 2020 flow diagram (Figure 1).

### 3.2. Study Characteristics

#### 3.2.1. ctDNA Studies

The 13 included ctDNA studies comprised 1632 evaluable patients (Table 1). They were published between 2015 and 2026 and conducted in Canada (n = 4), the United States (n = 3), the United Kingdom (n = 3), Belgium, China, and Taiwan, with sample sizes ranging from 23 to 712. Four studies were secondary analyses of RCTs, eight were prospective cohort studies, and one was retrospective. The included populations consisted of patients with stages I-III early breast cancer, triple-negative breast cancer, HER2-positive early breast cancer, hormone receptor-positive/HER2-negative disease, and mixed-subtype early breast cancer cohorts.

ctDNA detection platforms were heterogeneous, including tumor-informed PCR-based assays (n = 3), Signatera (n = 3), tissue-free epigenomic methylation-based assays (n = 3), ultra-deep personalized sequencing (n = 1), targeted ddPCR/NGS panels (n = 1), and personalized tumor-specific NGS (n = 2). Four studies reported post-adjuvant and nine reported post-neoadjuvant chemotherapy. By reported outcomes, five studies evaluated RFS, two evaluated EFS, three evaluated DRFS/DRFI, and one each evaluated RFI, PFS, and metastatic recurrence. 

Median follow-up ranged from 24 months (Li S et al., 2025) [28] to 77 months (Shaw et al., 2024) [29]. Seven studies reported multivariate HRs and six reported UV HRs only. The included studies varied substantially in patient populations, ctDNA assays, and measurement timing, which may contribute to the potential heterogeneity.

#### 3.2.2. CTC Studies

A total of four studies comprising 1398 patients were included in the quantitative synthesis of CTC studies (Table 2). The studies were published between 2007 and 2020 and were conducted in Greece, the United States, Germany, and South Korea. Three studies were prospective cohort studies, while one represented a secondary analysis of an RCT. The included populations consisted of patients with stages I-III early breast cancer, high-risk early breast cancer, and triple-negative breast cancer. Sample sizes ranged from 40 patients (Gwark et al., 2020) [30] to 1087 patients (Trapp et al., 2019) [31].

CTC detection platforms varied across studies, including nested RT-PCR for HER2 mRNA (n = 1), the FDA-approved CellSearch system (EpCAM+/CK+/CD45−; n = 2), and the SMART BIOPSY immunofluorescence platform (n = 1). Post-treatment MRD time points comprised post-NACT (n = 2) and post-adjuvant chemotherapy (n = 2). Reported outcomes included DFI, DFS, RFS, and RFI. Median follow-up duration ranged from 30 months (Hall et al., 2015) [32] to 72 months (Apostolaki et al., 2007) [33]. All four studies reported multivariate Cox regression HRs.

**Table 1:** Main characteristics of the included ctDNA studies.

First Author (Year)	Country	Study Design	N	Patient Population	ctDNA Detection Method	Timing	Cut-Off	Outcome	Median Follow-Up (mo.)	Adjustment
Garcia-Murillas (2015) [34]	UK	Prospective	55	Stages I–III; all subtypes	Personalized dPCR (mutation-specific)	Post-adj	Any ctDNA	RFS	~24–36	UV (Cox)
Cavallone (2020) [35]	Canada	RCT (secondary)	23	TNBC	Personalized ddPCR (WES-guided)	Post-NACT	Per-variant threshold	RFS	63	UV (Cox)
Cailleux (2022) [36]	Belgium	Prospective	44	HR+/TNBC/HER2+	Signatera (Natera)	Post-NACT	Any ctDNA	EFS	36	MV (adj pCR)
Ademuyiwa (2025) [37]	USA	RCT (secondary)	55	Stages II–III TNBC	Epigenomic (Guardant Reveal)	Post-NACT	Any ctDNA	RFI	50	MV (Cox)
Elliott (2025b) [38]	Canada	Prospective	95	ER+/TNBC early BC	Epigenomic mMRD (Guardant Reveal)	Post-NACT	Methylation ctDNA	EFS	35	MV (Cox)
Lin (2025) [39]	Taiwan	Prospective	117	HER2+ early BC	Tumor-informed NGS (HER2+-specific)	Post-NACT	Any ctDNA	RFS	~48	MV (Cox)
Coombes (2019) [24]	UK	Prospective	49	Non-metastatic BC	Personalized ultra-deep seq (Natera precursor)	Post-adj	≥2 variants	RFS	Up to 48	UV (Cox)
Zaikova (2024) [27]	Canada	Prospective	130	Non-metastatic TNBC	ddPCR + NGS hotspot panel	Post-adj/post-tx	≥1 variant (VAF ≥1%)	PFS	25	MV
Shaw (2024) [29]	UK	Prospective	156	Mixed; HR+-dominant	Signatera (Natera)	Post-adj	≥2 variants	RFS	77	MV (Cox)
Elliott (2025) [40]	Canada	Retrospective	34	Stages I–III; all subtypes	SV-based dPCR (WGS-guided)	Post-NACT	≥1 structural variant	DRFI	40	UV
Li S (2025) [28]	China	Prospective	118	Stages II–III TNBC	Tumor-informed NGS (Geneplus)	Post-NACT	≥1 tumor-specific variant	DRFS	24	UV
Magbanua (2025) [41]	USA	RCT (secondary)	712	NAT-resistant early BC (I-SPY2)	Signatera (Natera)	Post-NACT	≥2 variants	Met. recurrence	56	MV (Cox)
Grinshpun (2026) [42]	USA	RCT (secondary)	44	HR+ HER2−; Stages I–III	Epigenomic (Guardant Reveal)	Post-NACT	Any ctDNA	DRFI	35	UV

Abbreviations: BC, breast cancer; ctDNA, circulating tumor DNA; ddPCR, droplet digital polymerase chain reaction; dPCR, digital polymerase chain reaction; DRFI, distant recurrence-free interval; DRFS, distant recurrence-free survival; EFS, event-free survival; ER, estrogen receptor; HER2, human epidermal growth factor receptor 2; HR, hormone receptor; mMRD, methylation-based minimal residual disease; mo., months; MV, multivariable; NACT, neoadjuvant chemotherapy; NAT, neoadjuvant therapy; NGS, next-generation sequencing; pCR, pathological complete response; PFS, progression-free survival; RCT, randomized controlled trial; RFI, relapse-free interval; RFS, recurrence-free survival; SV, structural variant; TNBC, triple-negative breast cancer; UV, univariable; VAF, variant allele frequency; WES, whole-exome sequencing; WGS, whole-genome sequencing.

**Table 2:** Main characteristics of the included CTC studies.

First Author (Year)	Country	Study Design	N	Patient Population	CTC Detection Method	CTC Marker	Timing	Cut-Off	Outcome	Follow-Up (mo.)	Adjustment
Apostolaki (2007) [33]	Greece	Prospective	214	Stages I-II; early BC	Nested RT-PCR	HER2 mRNA	Post-adjuvant	Presence vs absence	DFI	72 (5–108)	MV (Cox)
Hall (2015) [32]	USA	Prospective	57	Stages I-III TNBC	CellSearch	EpCAM+/CK+/CD45−	Post-NACT	≥1 CTC/7.5 mL	RFS	30	MV (Cox)
Trapp (2019) [31]	Germany	RCT (secondary)	1087	Stages I-III high-risk early BC	CellSearch	EpCAM+/CK+/CD45−	Post-adjuvant	≥1 CTC/7.5 mL	DFS	37	MV (Cox)
Gwark (2020) [30]	South Korea	Prospective	40	Stages I-III TNBC	SMART BIOPSY	CK+/EpCAM+/CD45−	Post-NACT	≥5 CTCs	RFS	37.3	MV (Cox)

Abbreviations: BC, breast cancer; CD45, cluster of differentiation 45 (leukocyte common antigen); CK, cytokeratin; CTC, circulating tumor cell; DFI, disease-free interval; DFS, disease-free survival; EpCAM, epithelial cell adhesion molecule; HER2, human epidermal growth factor receptor 2; HR, hormone receptor; MV, multivariable; NACT, neoadjuvant chemotherapy; RCT, randomized controlled trial; RFS, recurrence-free survival; RT-PCR, reverse transcription polymerase chain reaction; TNBC, triple-negative breast cancer.

### 3.3. Prognostic Value of ctDNA

#### 3.3.1. Primary Meta-Analysis of ctDNA

**Figure 2:**
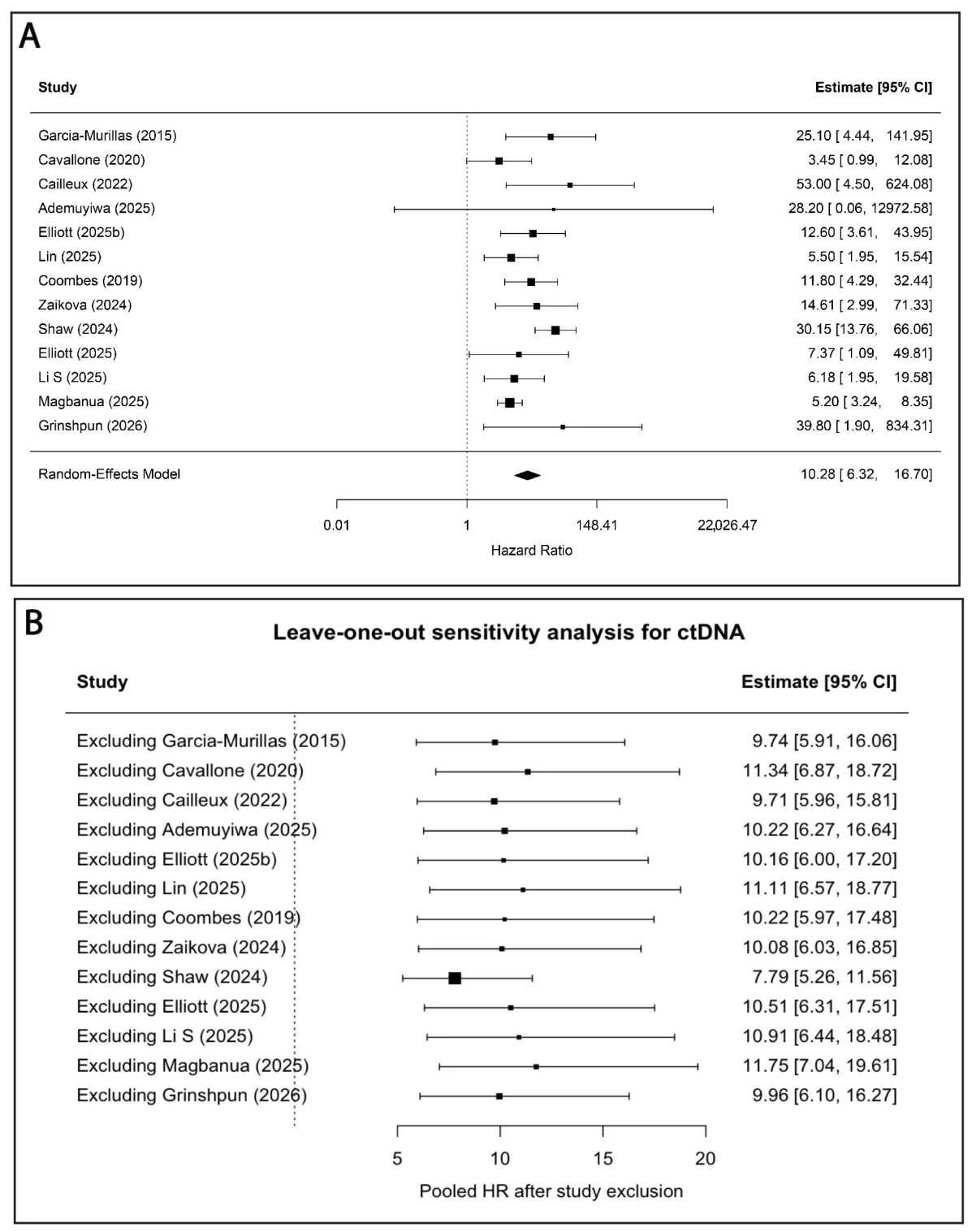
Prognostic significance and sensitivity analysis of post-treatment ctDNA positivity in early breast cancer. (**A**) Forest plot showing the association between post-treatment ctDNA positivity and recurrence-related outcomes in patients with early BC. Hazard ratios (HRs) and 95% CIs from 13 studies were pooled using a random-effects model. Squares represent individual study estimates, with square size proportional to study weight, and horizontal lines indicate 95% CIs. The diamond represents the pooled effect estimate. Post-treatment ctDNA positivity was significantly associated with an increased risk of recurrence or adverse clinical outcomes (pooled HR = 10.28, 95% CI: 6.32–16.70). Moderate heterogeneity was observed across studies (I^2^ = 47.3%, τ^2^ = 0.31, Q = 23.09, *p* = 0.027). (**B**) Leave-one-out sensitivity analysis of the ctDNA meta-analysis. Sequential exclusion of individual studies produced pooled HRs ranging from 7.79 to 11.75, with all estimates remaining statistically significant. No single study substantially influenced the overall effect estimate, supporting the robustness of the association between post-treatment ctDNA positivity and adverse clinical outcomes. The largest reduction in heterogeneity was observed after exclusion of Shaw et al., 2024 [29], although the pooled effect remained strong and statistically significant.

The primary ctDNA meta-analysis included all 13 eligible studies (seven MV and six UV) and used a random-effects model, with UV and MV HRs pooled as the primary estimate. Post-treatment ctDNA positivity was significantly associated with poorer survival outcomes in patients with early breast cancer (pooled HR = 10.28, 95% CI: 6.32–16.70, *p* < 0.0001) (Figure 2A). Moderate between-study heterogeneity was observed (I^2^ = 47.3%, τ^2^ = 0.311), and the Q-test indicated statistically significant heterogeneity (Q = 23.09, *p* = 0.027). Despite this variability, the prognostic effect of ctDNA remained consistently strong across studies. Study weights reflected the precision of the individual estimates: Magbanua et al., 2025 [41], Shaw et al., 2024 [29], and Coombes et al., 2019 [24] contributed most (16.6%, 13.0%, and 10.6%, respectively), whereas Ademuyiwa 2025 contributed negligibly (0.6%) because of an extremely wide confidence interval (0.06–12,700) arising from very few ctDNA-positive cases (Supplementary Table S3).

#### 3.3.2. Subgroup Analyses of ctDNA

Subgroup analysis demonstrated that the prognostic effect of ctDNA positivity varied significantly according to the timing of assessment (QM = 13.54, *p* = 0.0002). The association was strongest when ctDNA was measured after adjuvant therapy (HR = 20.62, 95% CI: 11.93–35.66), whereas a weaker but still significant effect was observed following neoadjuvant chemotherapy (HR = 6.07, 95% CI: 4.26–8.64). Timing accounted for all observed between-study heterogeneity (R^2^ = 100%), with no residual heterogeneity remaining after subgroup stratification (I^2^ = 0%, QE *p* = 0.57) (Table 3). This indicates that the timing of ctDNA assessment was a major source of variability across the included studies. However, the post-adjuvant subgroup comprised only four studies, and an R2 of 100% estimated from 13 studies is itself imprecise; this subgroup finding should therefore be regarded as hypothesis-generating rather than definitive.

Subgroup analysis based on breast cancer subtype showed no statistically significant differences in effect estimates (QM = 4.49, *p* = 0.21), suggesting that the prognostic value of ctDNA is consistent across molecular subtypes. Although a higher hazard ratio was observed in hormone receptor-positive disease, this estimate was derived from a single study and should be interpreted with caution (Table 3). While subtype accounted for a proportion of between-study variability (R^2^ = 47%), the moderator effect was not statistically significant, and residual heterogeneity remained low (I^2^ = 25.11%, QE *p* = 0.25) (Table 3).

Similarly, no significant difference was found between studies reporting MV and UV hazard ratios (QM = 0.25, *p* = 0.62), and adjustment status explained none of the observed between-study heterogeneity (R^2^ = 0%; residual I^2^ = 49.32%), indicating that the prognostic significance of ctDNA positivity was not materially affected by the type of statistical analysis. Finally, three exploratory meta-regression analyses examining methodological heterogeneity within the ctDNA studies were conducted. No significant subgroup differences were found according to assay design (tumor-informed versus tumor-agnostic: QM = 0.64, *p* = 0.42), detection chemistry (mutation-based versus methylation-based: QM = 0.46, *p* = 0.50), or MRD-positivity definition (any detectable ctDNA or ≥1 variant versus ≥2 variants: QM = 0.10, *p* = 0.75). None of these moderators explained between-study variance (R^2^ = 0%), and residual heterogeneity remained moderate (I^2^ = 48.64–49.66%). Given the small subgroup sizes and wide confidence intervals, these findings should be considered exploratory and not interpreted as evidence of equivalence between assay technologies (Supplementary Table S4).

### 3.4. Sensitivity Analyses of ctDNA

Leave-one-out sensitivity analysis demonstrated the robustness of the pooled ctDNA effect estimate. Excluding individual studies one at a time produced pooled HRs ranging from 7.79 to 11.75, all highly significant (*p* < 0.0001), with heterogeneity remaining moderate in most iterations (I^2^ 35.2–50.9%). The lowest estimate followed the exclusion of Shaw et al., 2024 [29] which also markedly reduced heterogeneity (I^2^ = 15.3%), and the highest followed the exclusion of Magbanua et al., 2025 [41] the largest study (Figure 2B; Supplementary Table S1). Excluding both studies identified as major contributors to heterogeneity (Shaw et al., 2024 [29] and Grinshpun et al., 2026 [42]) gave a comparable result (Table 4). The prognostic value of ctDNA was therefore stable across studies and was not driven by any individual dataset.

A fixed-effects model produced a slightly lower pooled estimate (HR = 8.69, 95% CI: 6.46–11.70); given the moderate between-study heterogeneity, the random-effects model was retained as the primary analysis. Sensitivity analysis restricted to studies reporting MV HRs demonstrated a similarly strong association between ctDNA positivity and adverse outcomes (HR = 11.84, 95% CI: 5.72–24.52; *p* < 0.001), indicating that the prognostic value of ctDNA remained significant after adjustment for potential confounding factors. However, substantial heterogeneity was observed (I^2^ = 62.5%), reflecting variability among study designs and analytical approaches (Table 4). Collectively, these results support that post-treatment ctDNA is a powerful but methodologically heterogeneous biomarker.

### 3.5. Prognostic Value of CTCs 

#### 3.5.1. Primary Analyses of CTCs

**Figure 3:**
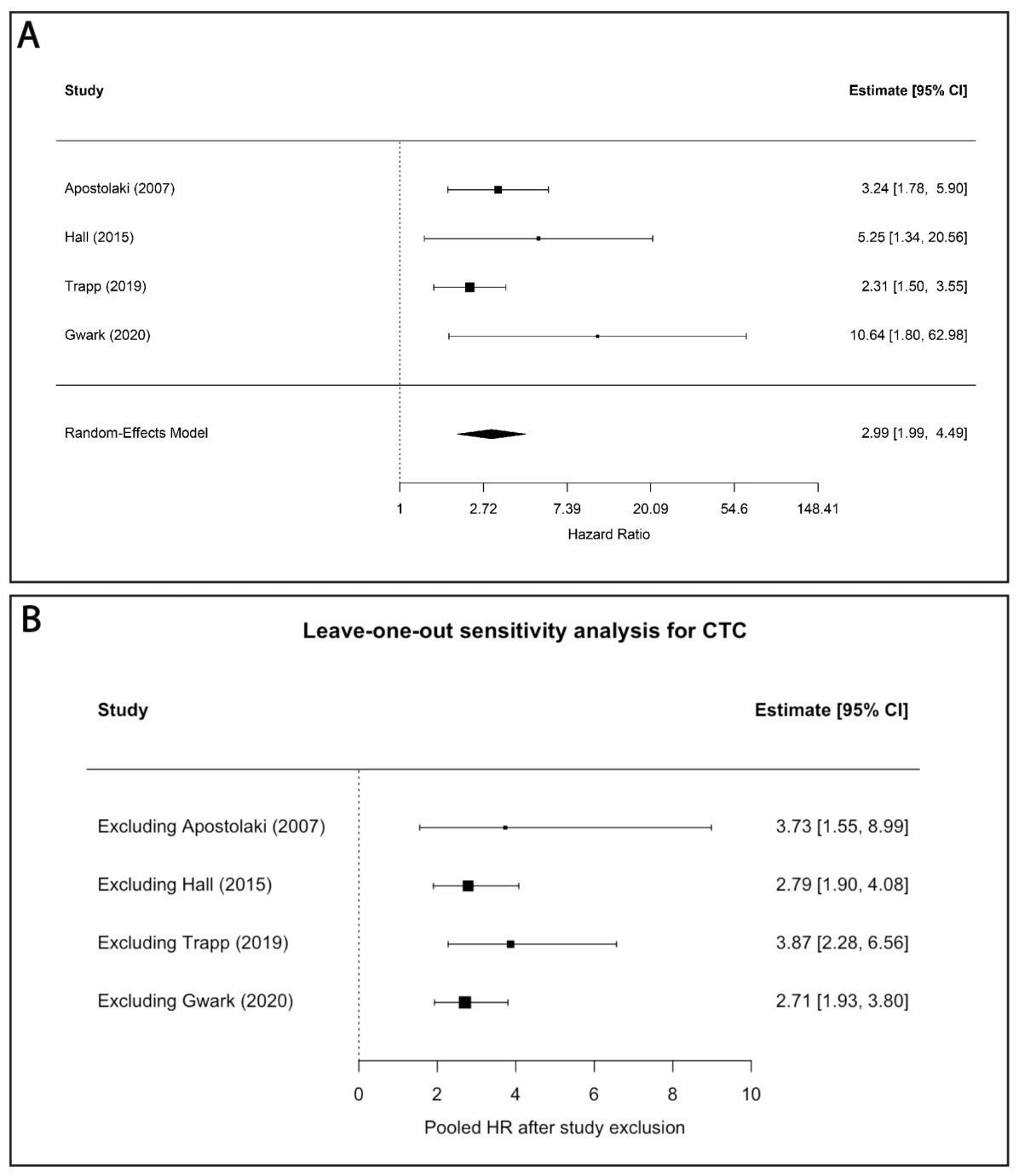
Prognostic significance and sensitivity analysis of post-treatment circulating tumor cells (CTCs) in early breast cancer. (**A**) Forest plot showing the association between post-treatment CTC positivity and recurrence-related outcomes in patients with early BC. HRs and 95% CIs from four studies [30,31,32,33] were pooled using a random-effects model. Squares represent individual study estimates, with square size proportional to study weight, and horizontal lines indicate 95% CIs. The diamond represents the pooled effect estimate. Post-treatment CTC positivity was significantly associated with an increased risk of recurrence or adverse clinical outcomes (pooled HR = 2.99, 95% CI: 1.99–4.49). Low between-study heterogeneity was observed (I^2^ = 17.2%, τ^2^ = 0.03, Q = 3.96, *p* = 0.27), indicating a consistent prognostic effect across the included studies. (**B**) Leave-one-out sensitivity analysis of the CTC meta-analysis. Sequential exclusion of individual studies demonstrated a stable pooled effect estimate, with hazard ratios ranging from 2.71 to 3.87. The overall association between post-treatment CTC positivity and increased recurrence risk remained significant in all analyses, indicating that no single study had a major impact on the pooled result. These findings support the robustness of the primary meta-analysis, although interpretation should be cautious because only four studies were included.

Four studies involving 1398 patients with early breast cancer were included in the CTC meta-analysis. Random-effects meta-analysis demonstrated that post-treatment CTC positivity was associated with a significantly increased risk of recurrence (pooled HR = 2.99, 95% CI: 1.99–4.49, *p* < 0.0001) (Figure 3A). Between-study heterogeneity was low (I^2^ = 17.2%, τ^2^ = 0.033) and not statistically significant (Q = 3.96, *p* = 0.27), indicating consistent prognostic effects across studies. The largest contribution to the pooled estimate was from Trapp et al., 2019 [31], which accounted for 52.8% of the total study weight, followed by Apostolaki et al., 2007 [33] (33.9%), Hall et al., 2015 [32] (8.3%), and Gwark et al., 2020 [30] (5.0%) (Supplementary Table S5).

#### 3.5.2. Subgroup Analyses of CTCs

Subgroup analysis according to the timing of CTC assessment demonstrated a stronger prognostic effect of CTC positivity in studies evaluating patients after neoadjuvant chemotherapy compared with those assessed after adjuvant therapy (HR = 6.82, 95% CI: 2.31–20.15) vs. (HR = 2.6, 95% CI: 1.83–3.68, respectively). However, the difference between subgroups was not statistically significant (QM = 2.78, *p* = 0.096). After stratification by timing, no residual heterogeneity remained (I^2^ = 0%, QE = 1.18, *p* = 0.553), suggesting that timing may contribute to between-study variability (Table 5). Nevertheless, these findings should be interpreted cautiously because only four studies were available for analysis. Subgroup analysis by breast cancer subtype yielded identical estimates (QM = 2.78, *p* = 0.096; Table 5) because all post-neoadjuvant CTC studies were conducted in TNBC cohorts; timing and subtype are therefore completely confounded in this pool, and neither subgroup analysis can be interpreted independently. An exploratory comparison found no significant difference between studies using CellSearch and those using other CTC detection platforms (QM = 0.46, *p* = 0.50; R^2^ = 0%; Supplementary Table S4). This analysis was based on only two studies per subgroup and was therefore substantially underpowered.

#### 3.5.3. Sensitivity Analyses of CTCs

Leave-one-out sensitivity analysis demonstrated the stability of the pooled estimate of the CTC effect. Excluding individual studies one at a time produced pooled hazard ratios ranging from 2.71 (excluding Gwark et al., 2020) [30] to 3.87 (excluding Trapp et al., 2019) [31], all statistically significant (Figure 3B; Supplementary Table S6). A fixed-effects model gave a consistent result (HR = 2.84, 95% CI: 2.04–3.96). No single study had a substantial impact on the pooled estimate, supporting the robustness of the association between post-treatment CTC positivity and increased recurrence risk.

### 3.6. Publication Bias

**Figure 4:**
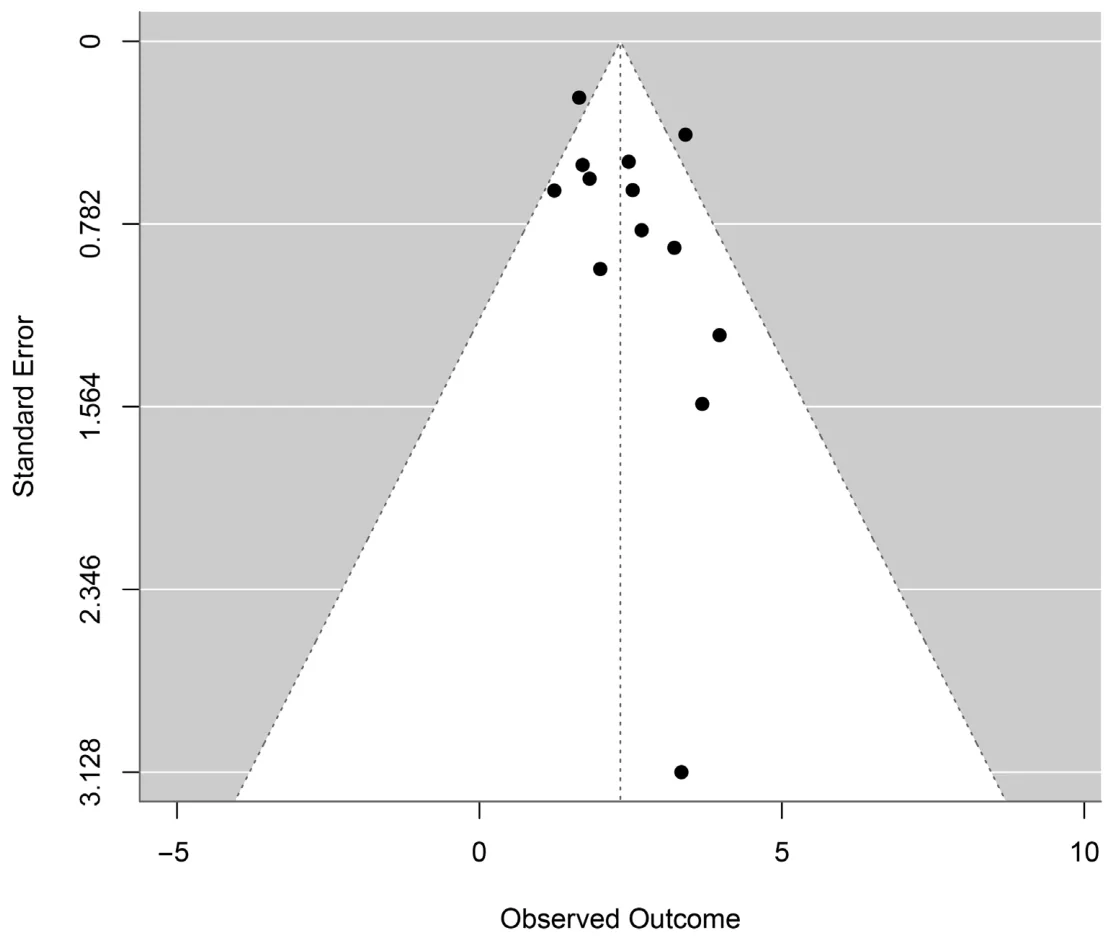
Funnel plot of the included ctDNA studies. Each point represents an individual study. The distribution of studies was generally symmetrical around the pooled effect estimate, suggesting no obvious evidence of publication bias or small-study effects. This observation is supported by Egger’s regression test, which showed no statistically significant asymmetry in the funnel plot (*p* = 0.21).

Visual inspection of the funnel plot did not reveal marked asymmetry among the included ctDNA studies (Figure 4). Egger’s regression test showed no evidence of publication bias for ctDNA studies (z = 1.27, *p* = 0.205). The limit estimate remained statistically significant, suggesting that the observed association is unlikely to be explained by small-study effects. However, these findings should be interpreted with caution due to the moderate heterogeneity among the included studies (I^2^ = 47.3%). For CTC studies, formal assessment was not performed due to the limited number of included studies (k = 4).

### 3.7. Quality Assessment (Risk of Bias)

Across all 17 included studies, the overall risk of bias assessed using the QUIPS tool was low in three studies (18%), moderate in eleven studies (65%), and high in three studies (18%) (Supplementary Table S7). No study was rated as having a high risk of bias for study participation or outcome measurement, indicating that most studies included well-defined patient cohorts and standardized recurrence-related outcomes assessed through appropriate follow-up. 

In contrast, study attrition (13/17 moderate, 1/17 high) and study confounding (7/17 moderate, 3/17 high) were the domains with the greatest risk of bias. Attrition concerns most often reflected the exclusion of patients who lacked sufficient tumor tissue, detectable somatic mutation, or paired serial blood samples required for ctDNA analysis. For example, in Cavallone et al., 2020 [35], only 26 of 60 patients (43%) from the parent Q-CROC-03 trial cohort were eligible for the analysis. Confounding concerns were primarily due to the use of univariate analyses without adjustment for potential confounders (Cavallone et al., 2020 [35]; Grinshpun et al., 2026 [42]) or the use of underpowered, subgroup-restricted multivariable models (Gwark et al., 2020 [30]).

Three studies were judged to have an overall low risk of bias, Shaw et al., 2024 [29], Elliott et al., 2025b [38]; epigenomic mMRD/LIBERATE cohort), and Magbanua et al., 2025 [41]; I-SPY2), each characterized by large or well-characterized cohorts, transparent reporting of attrition, validated assay platforms, and multivariable-adjusted analyses. In contrast, three studies (Gwark, et al., 2020 [30]; Cavallone et al., 2020 [35]; Grinshpun et al., 2026 [42]) were rated as having an overall high risk of bias, mainly because of small sample sizes, retrospective or post hoc analyses, a limited number of recurrence events, and limited or absent adjustment for multivariable confounders.

## 4. Discussion

This systematic review and meta-analysis is, to our knowledge, the first to simultaneously evaluate ctDNA and CTCs as post-treatment markers of MRD in early breast cancer. Our findings demonstrated that both biomarkers were significantly associated with poorer survival outcomes following systemic therapy. CTC positivity was associated with an approximately threefold increase in recurrence risk (pooled HR 2.99, 95% CI: 1.99–4.49; k = 4; all MV; I^2^ = 17.20%), while ctDNA positivity was associated with a markedly stronger effect (pooled HR = 10.28; 95% CI: 6.32–16.70; k = 13; UV&MV; I^2^ = 47.32%). Overall, the pooled prognostic association was approximately 3.4-fold stronger for ctDNA than for CTCs. However, this was an indirect comparison because no included study evaluated both biomarkers in the same patients. In addition, the ctDNA and CTC studies differed in publication period, patient populations, treatment settings, outcome definitions, and assay methods. Therefore, these findings should not be interpreted as evidence that ctDNA outperforms CTCs when the two are measured concurrently in the same patients. Across the 17 studies (3030 patients), post-treatment positivity for either biomarker was associated with an increased risk of recurrence. Notably, ctDNA positivity in particular was associated with a more than 10-fold increase in recurrence risk, underscoring its potential value for identifying persistent molecular residual disease. 

The stronger pooled prognostic association observed for ctDNA than for CTCs, although derived from an indirect comparison, is biologically plausible. One possible explanation is the higher analytical sensitivity of modern ctDNA assays compared with morphological or mRNA-based CTC detection. CTCs are rare intact tumor cells that must survive in the bloodstream despite both mechanical and immune-mediated clearance. As a result, their detection in the adjuvant setting often occurs at very low levels (typically one to five cells per 7.5 mL blood), approaching the sensitivity limits of currently validated platforms such as CellSearch [43]. In contrast, ctDNA is continuously released into the circulation from apoptotic tumor cells and can be detected at extremely low concentrations using highly sensitive, tumor-informed sequencing approaches [19]. This greater sensitivity may allow ctDNA to identify residual disease more accurately, contributing to the stronger prognostic effect observed in our analysis.

In addition to differences in analytical sensitivity, ctDNA and CTCs may reflect different aspects of residual disease biology. CTCs are viable tumor cells capable of establishing distant metastases; therefore, their detection after treatment suggests the persistence of therapy-resistant cancer cells [18]. ctDNA, on the other hand, reflects the overall burden of residual disease and captures signals from multiple sites, including clinically undetectable micro-metastases. Furthermore, ctDNA can provide real-time information on tumor evolution and the emergence of treatment-resistant genetic alterations [44]. Importantly, both ctDNA and CTCs have been shown to predict recurrence independently of standard clinicopathological factors. This indicates that these biomarkers provide potentially complementary information about MRD beyond that obtained from conventional risk assessment approaches.

The strongest prognostic effect was observed for ctDNA assessed after adjuvant therapy (HR = 20.62; 95% CI: 11.93–35.66; I^2^ = 0%; k = 4), although this subgroup comprised only four studies and the estimate should be considered hypothesis-generating. This subgroup included studies that evaluated ctDNA during post-treatment surveillance, months to years after completion of all primary chemotherapy [24,27,29,34]. The detection of ctDNA after the completion of all planned therapy likely indicates the presence of persistent molecular residual disease despite apparently successful treatment and is therefore associated with a particularly high risk of recurrence. In contrast, ctDNA positivity detected after neoadjuvant chemotherapy was associated with a lower, although still substantial, risk of recurrence (HR = 6.07, 95% CI: 4.26–8.64, I^2^ = 0%; k = 9). This difference may reflect the biological distinction between residual disease detected during treatment and persistent MRD identified after completion of all curative-intent therapy. Together, these findings suggest that ctDNA assessment after completion of therapy may provide the greatest prognostic value for identifying patients at the highest risk of relapse, a hypothesis that requires confirmation in larger post-adjuvant cohorts.

Another important finding was the strong prognostic performance of tissue-free epigenomic ctDNA platforms, such as Guardant Reveal. Unlike tumor-informed assays, which require tumor tissue sequencing and the development of a personalized assay for each patient [45], tissue-free epigenomic approaches detect cancer-associated methylation patterns directly from plasma. This approach eliminates the need for prior tumor tissue analysis and may make MRD testing easier to implement in routine clinical practice. Although no significant differences between detection methods were identified in our meta-analysis, the high effect estimates reported for epigenomic assays suggest that they may represent a practical and scalable approach for post-treatment MRD assessment. If validated in larger prospective studies, tissue-free epigenomic ctDNA testing could support the wider adoption of MRD-guided surveillance strategies in early breast cancer.

For CTCs, the most robust evidence came from the SUCCESS A RCT sub-study by Trapp et al., 2019 [31], which included 1087 patients and was the largest CTC study in our analysis. It reported a 2.3-fold increase in the risk of recurrence in patients with detectable CTCs after treatment [31]. Because of its large sample size and its status as the only biomarker study planned and conducted within a large RCT, it contributed significantly to the overall pooled estimate. The moderate effect size observed in this study suggests that although CTC positivity is a clinically important predictor of recurrence, its prognostic impact may be less pronounced than that reported in some earlier, smaller studies [30,33]. Ongoing prospective clinical trials, including DETECT and CirCe01, are expected to clarify whether treatment decisions based on CTC status can improve outcomes for patients with breast cancer [16].

Previous ctDNA meta-analyses reported pooled HRs ranging from 6.92 to 8.17 for recurrence-related outcomes [46,47]. Our pooled estimate of 10.28 is broadly consistent with these earlier findings but is based on a larger and more up-to-date evidence base comprising 13 eligible studies. In addition, our analysis included several recently developed ctDNA technologies, particularly tissue-free epigenomic assays, as well as established tumor-informed approaches. This allowed for a more comprehensive assessment of the current landscape of ctDNA-based MRD detection in early breast cancer. Similarly, previous meta-analyses of CTCs in early breast cancer have reported pooled HRs ranging from 1.78 to 3.85 for recurrence-related outcomes [48,49]. Our pooled estimate of 2.99 falls within this range and is consistent with the findings of higher-quality prior analyses. Heterogeneity in our four-study CTC pool was lower (I^2^ = 17.2%) than the I^2^ of approximately 50–70% reported in earlier reviews, most likely because of our stricter selection criteria and focus on clearly defined post-treatment MRD assessment. 

For ctDNA, moderate heterogeneity was observed in the primary analysis (I^2^ = 47.32%), compared with the high heterogeneity (I^2^ = 79.0%) reported in previous meta-analyses [46,47]. This may reflect the more homogeneous study population and stricter study selection criteria used in the present review. Subgroup analysis by the timing of MRD assessment completely resolved this heterogeneity (I^2^ = 0% in both the post-NACT and post-adjuvant subgroups), suggesting that the timing of the ctDNA measurement was the main source of between-study variability in the ctDNA pool. Furthermore, similar effect estimates were observed in studies reporting multivariable-adjusted and univariable HRs (HR = 11.55 and HR = 8.91, respectively). This indicates that the association between post-treatment ctDNA positivity and adverse clinical outcomes was consistent across different study designs and statistical adjustments. In contrast, heterogeneity in the primary CTC meta-analysis was low (I^2^ = 17.2%; Q *p* = 0.27; τ^2^ = 0.033), indicating a consistent prognostic effect across the four included studies. 

The two biomarker pools are markedly asymmetric in size, comprising thirteen ctDNA studies and only four CTC studies. Although the same eligibility criteria were applied to both biomarkers, many CTC studies did not assess CTCs at a predefined post-treatment timepoint or report an extractable HR. Most large CTC cohorts focused principally on testing the prognostic value of CTCs at diagnosis or before chemotherapy, whereas ctDNA studies were more often designed specifically to evaluate post-treatment MRD. Several landmark CTC studies were therefore excluded because they reported only pre-treatment CTC status, used variable rather than protocol-defined sampling intervals, included cohorts already represented by an included study, or reported a null post-treatment association without an extractable effect estimate (Supplementary Table S2). This asymmetry reflects differences in the design of the two evidence bases rather than the differential application of the eligibility criteria. Consequently, the included ctDNA evidence was better aligned with our research question, whereas the pooled CTC estimate was based on a narrower and older subset of the available literature. Comparisons between the two pooled estimates should therefore be interpreted cautiously.

The QUIPS assessment indicated that the overall quality of the available evidence was generally moderate. The largest and most influential ctDNA studies (Shaw et al., 2024 [29]; Elliott et al., 2025b [38]; Magbanua et al., 2025 [41]) were rated as having a low risk of bias, and few studies were at high risk for study participation, prognostic factor, or outcome measurement, so the observed associations are unlikely to be explained by major methodological flaws. The main sources of bias, attrition and confounding, reflect the recognized challenges of MRD research: small cohorts, few recurrence events, and exclusion of patients lacking sufficient tumor tissue, detectable mutations, or adequate blood samples. Future studies would benefit from larger cohorts, prespecified multivariable analyses, and more transparent reporting of participant attrition.

This study has several strengths. To our knowledge, it is the first pre-registered systematic review and meta-analysis to evaluate ctDNA and CTCs as post-treatment MRD markers in early breast cancer within a single analytical framework. Careful study selection excluded overlapping cohorts and studies with limited extractable outcome data, improving comparability; risk of bias was assessed systematically with the QUIPS tool; and sensitivity analyses, together with the absence of significant funnel-plot asymmetry (*p* = 0.21), indicate that the results are stable across analytical approaches and not driven by any individual study. 

Several limitations should be acknowledged. Most importantly, the comparison between ctDNA and CTCs is indirect and is further affected by an asymmetry in how the two bodies of literature are represented here. The ctDNA studies included a full range of contemporary technologies (tumor-informed PCR and NGS, tissue-free epigenomic assays, ddPCR panels). On the other hand, only four CTC studies met the eligibility criteria; three of the four were published before 2020, and none used the newer CTC enrichment or characterization platforms. Any comparison between the pools is therefore also, in part, a comparison between the current generation of ctDNA assays and an earlier generation of CTC assays. Moreover, the pooled estimates were derived from entirely separate studies that differed in publication period (2007–2020 for CTCs versus 2015–2026 for ctDNA), patient populations, treatment settings, outcome definitions, and assay generation; no included study assessed both analytes in the same patients. The difference in pooled hazard ratios therefore cannot establish the superiority of one biomarker over the other, and studies measuring both biomarkers concurrently are required. 

Another important limitation is that recurrence-related endpoints (RFS, DFS, DFI, DRFS, DRFI, EFS, PFS, RFI, and metastatic recurrence) were combined into a single composite outcome. Although these endpoints all assess disease recurrence, they are not identical, and PFS differs conceptually from others. Combining them may have contributed to the observed heterogeneity and could bias pooled estimates in either direction. However, the leave-one-out analysis showed that no single endpoint had a major impact on the overall results. In addition, univariable and multivariable HRs were pooled in the primary analysis; the sensitivity analysis restricted to adjusted estimates gave a similar result (HR = 11.84), but residual confounding in the six univariable studies cannot be excluded. The post-adjuvant ctDNA subgroup comprised only four studies, so the marked difference from the post-neoadjuvant subgroup should be treated as hypothesis-generating. Publication bias and residual heterogeneity should also be considered. Although Egger’s test did not show significant publication bias in the ctDNA analysis (*p* = 0.21), all 13 included ctDNA studies reported positive associations, and no study reported null findings. Therefore, publication bias cannot be completely excluded, and the true effect may be smaller than the pooled estimate. Moderate heterogeneity was observed in the primary ctDNA analysis. Although subgroup analyses identified the timing of MRD assessment as a major source of variability, other factors, such as differences in treatment regimens, patient populations, assay sensitivity, and sample processing methods, may also have contributed to the observed heterogeneity. Finally, all included studies were observational, and no randomized clinical trials have yet demonstrated that treatment decisions guided by liquid biopsy findings improve patient outcomes. Therefore, while ctDNA and CTCs are strong prognostic markers, further prospective studies are needed to determine whether their use can improve clinical outcomes.

The present findings support the potential role of liquid biopsy-based MRD assessment in identifying patients at increased risk of recurrence after treatment for early breast cancer. In particular, the strong prognostic value of ctDNA suggests that post-treatment MRD assessment may help identify patients at very high risk of relapse who could benefit from closer follow-up or enrollment in MRD-guided clinical trials. However, further prospective studies are needed before ctDNA and CTC testing can be used to guide treatment decisions in routine clinical practice. In addition, the cost, availability, and standardization of ctDNA testing remain important considerations before widespread clinical implementation.

The findings of this meta-analysis highlight several areas for future research. First, prospective randomized trials are needed to determine whether treatment decisions guided by post-treatment ctDNA or CTC results can improve patient outcomes. Several ongoing trials, including ZEST (NCT04915755), TREAT-ABC, and c-TRAK TN2, currently address this question in specific patient populations. Second, direct comparisons of ctDNA assay platforms, particularly tumor-informed and tissue-free epigenomic approaches, in the same patient cohort are needed to determine the most effective approach for clinical use. Third, the combined use of ctDNA and CTCs as complementary MRD markers should be evaluated, as they may provide additional prognostic value when used together [50]. Fourth, the prognostic value of ctDNA detected during post-adjuvant surveillance should be validated in larger prospective studies. Finally, further research is needed to assess the role of serial ctDNA monitoring in detecting late recurrence, especially in hormone receptor-positive breast cancer, where residual disease may persist for many years after treatment.

Three implementation challenges also require attention. First, analytical standardization, including limits of detection, plasma input volume, variant-calling thresholds, and the definition of MRD positivity, differs substantially across platforms. Standardized reference materials and minimum reporting standards are needed before the results can be reliably compared or pooled across assays. Second, the cost-effectiveness of these technologies remains uncertain. Tumor-informed assays require tumor sequencing and personalized panel design, while tissue-free epigenomic assays may be more scalable. Both require formal economic evaluation in early breast cancer. Third, integration into clinical guidelines remains premature. Current ASCO and ESMO recommendations do not endorse routine ctDNA or CTC testing for MRD detection in early breast cancer outside clinical trials because randomized evidence that MRD-guided management improves patient outcomes is lacking. Ongoing MRD-guided trials should define the levels of analytical validity, clinical validity, and clinical utility needed to support future guideline recommendations.

## 5. Conclusions

Both post-treatment ctDNA and CTCs were significantly associated with poorer survival outcomes in early breast cancer, supporting their value as prognostic markers of minimal residual disease. ctDNA showed a stronger pooled prognostic association than CTCs (pooled HR = 10.28 vs. 2.99), which may relate to its higher analytical sensitivity for residual disease across multiple anatomical sites. This comparison is indirect, however, because the two estimates derive from separate sets of studies; direct head-to-head comparisons within the same cohorts are still required. The prognostic value of ctDNA remained consistent across different breast cancer subtypes, detection platforms, and study designs. In addition, ctDNA detected after the completion of systemic treatment identified the subgroup of patients at the highest risk of recurrence, although this observation rests on only four studies and is hypothesis-generating. Overall, these findings support the potential use of liquid biopsy-based MRD assessment to improve post-treatment risk stratification in early breast cancer. However, further prospective randomized studies are needed to determine whether MRD-guided treatment strategies can improve patient outcomes.

## Figures and Tables

**Table 3:** Subgroup analysis of 13 eligible studies of ctDNA.

Subgroup	k	Pooled HR (95%CI)	QM (Test of Moderator)	*p* (Effect)	Note
ctDNA subgroup: Timing of MRD Measurement					
Intercept (Post-adj)	4	20.62 (11.93–35.66)	—	<0.0001	Model heterogeneity: I^2^ = 0.0%, τ^2^ = 0.000, QE = 9.56, *p* = 0.5707, R^2^ = 100%
Post-NACT	9	6.07 (4.26–8.64)	—	<0.0001	
Between-group difference (ratio of HRs)	—	0.294 (0.15–0.57)	13.54	0.0002	
ctDNA subgroup: Breast Cancer Subtype					
Intercept (Mixed early BC)	4	8.09 (4.18–15.7)	—	<0.001	Model heterogeneity: I^2^ = 25.11%, τ^2^ = 0.1644, QE = 11.3684, *p*(QE) = 0.2513, R^2^ = 47.14%. Note: No HR reported as this is a multi-category moderator (four subtypes)
HER2-positive	2	7.33 (1.91–28.46)	—	0.89	
Hormone receptor-positive	1	24.96 (1.02–136.43)	—	0.047	
Triple-negative BC	6	8.86 (2.43–30.06)	—	0.85	
Between-group difference (ratio of HRs)	—	—	4.49	0.21	
ctDNA subgroup: Univariate vs Multivariate Analysis					
Intercept (MV)	7	11.55 (5.96–22.38)	—	<0.0001	Model heterogeneity: I^2^ = 49.32%, τ^2^ = 0.3606, QE = 23.083, *p* = 0.0172, R^2^ = 0%
UV	6	8.91 (4.08–19.44)	—		
Between-group difference (ratio of HRs)	—	0.77 (0.28–2.15)	0.25	0.62	
ctDNA subgroup: Detection Methods					
Intercept (Other methods)	9	10.89 (6.08–19.55)	—	—	Model heterogeneity: I^2^ = 50.60%, τ^2^ = 0.355, QE = 23.08, *p* = 0.017, R^2^ = 0%
PCR-based methods	4	8.94 (2.61–25.82)	—	—	
Between-group difference (ratio of HRs)	—	0.82 (0.26–2.58)	0.11	0.74	

Intercept means the reference group. Abbreviations: BC, breast cancer; CI, confidence interval; ctDNA, circulating tumor DNA; HER2, human epidermal growth factor receptor 2; HR, hazard ratio; I^2^, Higgins’ inconsistency statistic; k, number of studies; MRD, minimal residual disease; MV, multivariable; NACT, neoadjuvant chemotherapy; QE, residual heterogeneity statistic; QM, test of moderators; R^2^, proportion of between-study heterogeneity explained by the moderator; τ^2^, between-study variance; UV, univariable.

**Table 4:** Sensitivity analyses of the prognostic value of post-treatment ctDNA in early breast cancer.

Analysis	Pooled HR (95% CI)	*p*-Value	I^2^	Note
SENSITIVITY (Excluding problematic studies)				
All studies (REML)	10.28 (6.32–16.70)	<0.001	47%	Primary analysis
Excluding Shaw and Grinshpun	7.79 (5.25–11.55)	<0.001	15%	Heterogeneity markedly reduced
SENSITIVITY (Fixed Effects vs Random Effects Comparison)				
Random Effects (REML)	10.28 (6.32–16.70)	<0.0001	--	Primary analysis
Fixed Effects	8.69 (6.46–11.70)	<0.0001	47%	Moderate heterogeneity (I^2^ = 47%). Fixed-effects model produced narrower confidence intervals; however, due to moderate heterogeneity, random-effects preferred
SENSITIVITY (MV-only vs all studies)				
All studies (REML)	10.28 (6.32–16.70)	<0.001	47%	Primary analysis
Random (REML, MV-only)	11.84 (5.72–24.52)	<0.001	62%	Effect remains strong after adjustment

Abbreviations: CI, confidence interval; ctDNA, circulating tumor DNA; HR, hazard ratio; I^2^, Higgins’ inconsistency statistic; MV, multivariable; REML, restricted maximum-likelihood. Random-effects models were estimated using the REML method.

**Table 5:** Subgroup analysis of the four eligible studies of CTC.

Subgroup	k	Pooled HR (95%CI)	QM (Test of Moderator)	*p* (Effect)	Note
CTC SUBGROUP: Timing of MRD Measurement					
Intercept (post-adj)	2	2.59 (1.83–3.68)	—	<0.0001	Model heterogeneity: I^2^ = 0.0%, τ^2^ = 0.000, QE = 1.1848, *p* = 0.553, R^2^ = 100%
Post-NACT	2	6.82 (2.31–20.15)	—	0.1	
Between-group difference (ratio of HRs)	—	2.63 (0.84–8.22)	2.78	0.096	
CTC SUBGROUP: Breast Cancer Subtype					
Intercept (Mixed early BC)	2	2.59 (1.83–3.68)	—	<0.001	Model heterogeneity: I^2^ = 0.0%, τ^2^ = 0.0000, QE = 1.1848, *p*(QE) = 0.553, R^2^ = 47.14%
Triple-negative BC	2	6.82 (2.31–20.15)	—	0.1	
Between-group difference (ratio of hazard ratios)	—	2.63 (0.84–8.22)	2.78	0.0955	

Intercept means the reference group. Abbreviations: BC, breast cancer; CI, confidence interval; CTC, circulating tumor cell; HR, hazard ratio; I^2^, Higgins’ inconsistency statistic; k, number of studies; MRD, minimal residual disease; NACT, neoadjuvant chemotherapy; Post-adj, post-adjuvant therapy; QE, residual heterogeneity statistic; QM, test of moderators; R^2^, proportion of between-study heterogeneity explained by the moderator; τ^2^, between-study variance.

## Data Availability

The original contributions presented in this study are included in the article/supplementary material. Further inquiries can be directed to the corresponding author.

## Supplementary Materials

The following supporting information can be downloaded at: https://www.mdpi.com/article/10.3390/ijms27177719/s1.

## Abbreviations

The following abbreviations are used in this manuscript:

τ^2^	Between-study variance (tau-squared; estimated by REML)
adj CT	Adjuvant chemotherapy
BC	Breast cancer
cfDNA	Cell-free DNA
95% CI	95% confidence interval
CI	Confidence interval
CK	Cytokeratin
CK-19	Cytokeratin-19
CTC	Circulating tumor cell(s)
ctDNA	Circulating tumor DNA
ddPCR	Droplet digital polymerase chain reaction
DFI	Disease-free interval
DFS	Disease-free survival
dPCR	Digital polymerase chain reaction
DRFI	Distant recurrence-free interval
DRFS	Distant recurrence-free survival
EFS	Event-free survival
EpCAM	Epithelial cell adhesion molecule
ER	Estrogen receptor
HER2	Human epidermal growth factor receptor 2
HR	Hazard ratio
I^2^	Inconsistency statistic (quantitative measure of between-study heterogeneity)
k	Number of studies included in a meta-analytic pool
MRD	Minimal residual disease
MV	Multivariable analysis
NACT	Neoadjuvant chemotherapy
NET	Neoadjuvant/adjuvant endocrine therapy
NGS	Next-generation sequencing
OS	Overall survival
pCR	Pathological complete response
PCR	Polymerase chain reaction
PFS	Progression-free survival
PR	Progesterone receptor
PRISMA	Preferred Reporting Items for Systematic Reviews and Meta-Analyses
PROSPERO	International Prospective Register of Systematic Reviews
Q	Cochran’s Q statistic (test for between-study heterogeneity)
QE	Q statistic for residual heterogeneity (within a subgroup)
QM	Q statistic for test of moderator effect (between subgroups)
QUIPS	Quality In Prognosis Studies
RCT	Randomized controlled trial
REML	Restricted maximum-likelihood estimator (method for τ^2^ estimation)
RFI	Relapse-free interval
RFS	Recurrence-free survival
RT-PCR	Reverse transcriptase polymerase chain reaction
R^2^	Proportion of between-study heterogeneity explained by a moderator variable
SV	Structural variant
TNBC	Triple-negative breast cancer
UV	Univariable analysis
VAF	Variant allele frequency
WES	Whole-exome sequencing
